# Hippocampal Mitochondrial Abnormalities Induced the Dendritic Complexity Reduction and Cognitive Decline in a Rat Model of Spinal Cord Injury

**DOI:** 10.1155/2022/9253916

**Published:** 2022-05-04

**Authors:** Xvlei Hu, Liang Wu, Yujun Wen, Juan Liu, Hailiang Li, Yifan Zhang, Zhihua Wang, Jiangwei Ding, Zhong Zeng, Hechun Xia

**Affiliations:** ^1^Department of Neurosurgery, General Hospital of Ningxia Medical University, Yinchuan, China; ^2^Ningxia Key Laboratory of Craniocerebral Diseases, Yinchuan, China; ^3^School of Clinical Medicine, Ningxia Medical University, Yinchuan, China; ^4^School of Basic Medical Sciences, Ningxia Medical University, Yinchuan, China; ^5^Ningxia Human Stem Cell Research Institute, General Hospital of Ningxia Medical University, Yinchuan, China

## Abstract

Spinal cord injury (SCI) is a progressive neurodegenerative disease in addition to a traumatic event. Cognitive dysfunction following SCI has been widely reported in patients and animal models. However, the neuroanatomical changes affecting cognitive function after SCI, as well as the mechanisms behind these changes, have so far remained elusive. Herein, we found that SCI accelerates oxidative stress damage of hippocampal neuronal mitochondria. Then, for the first time, we presented a three-dimensional morphological atlas of rat hippocampal neurons generated using a fluorescence Micro-Optical Sectioning Tomography system, a method that accurately identifies the spatial localization of neurons and trace neurites. We showed that the number of dendritic branches and dendritic length was decreased in late stage of SCI. Western blot and transmission electron microscopy analyses also showed a decrease in synaptic communication. In addition, a battery of behavioral tests in these animals revealed hippocampal based cognitive dysfunction, which could be attributed to changes in the dendritic complexity of hippocampal neurons. Taken together, these results suggested that mitochondrial abnormalities in hippocampal neurons induced the dendritic complexity reduction and cognitive decline following SCI. Our study highlights the neuroanatomical basis and importance of mitochondria in brain degeneration following SCI, which might contribute to propose new therapeutic strategies.

## 1. Introduction

Spinal cord injury (SCI) is a significant global public health issue [1] and a major cause of lost human potential. Further, SCI imposes a huge financial and emotional burden on families and societies. Previous studies mostly focused on the sensorimotor systems [2–6], neuropathic pain [7–9], and autonomic dysfunctions [10] associated with SCI. However, SCI also causes serious psychological health problems. Clinical evidence show that SCI patients perform worse on attention, long-term memory, short-term memory, executive functions, processing speed, and learning ability [11–14]. In addition, these patients are more prone to anxiety and depression disorders [15–17]. The risk of cognitive impairment was up to 13 times higher in people with SCI than in healthy individuals [18]. Similar results were observed in the animal models [19–23]. Cognitive dysfunction is a serious secondary complication of SCI which hinders the process of physical rehabilitation [24, 25]. However, neuroanatomical changes affecting cognitive function after spinal cord injury and the mechanisms behind it remain unclear.

SCI has recently been recognized as a progressive neurodegenerative disease [26–28] and has been shown to cause atrophy and reorganization above the level of the injury, including sensorimotor cortex, corticospinal tracts, thalamus, and internal capsule [29–32]. Moreover, these structural and/or functional changes are not limited to the sensorimotor pathways but include other brain areas without such direct connections [33]. SCI-induced neuronal loss, changed inflammatory, and reduced neurogenesis in the hippocampus were observed in the rodent experiments [20, 21, 34].

Mitochondria are the main source of energy, supplying 90% of cellular energy via oxidative phosphorylation (OXPHOS) [35], which is also responsible for promoting the cellular stress response [36]. Neurons are particularly vulnerable to oxidative stress because of the high polyunsaturated fatty acid content in neuronal membranes, the significant oxygen consumption, and weak antioxidant defense [37]. Therefore, it is not surprising that mitochondrial abnormality was involved in neurodegenerative diseases, including Alzheimer's disease [38, 39], Parkinson's disease [40], amyotrophic lateral sclerosis [41], spinal muscular atrophy [42], and Huntington's disease [43]. Interestingly, mitochondrial dysfunction is one of the initiating events in many pathologies [44]. These data suggest that mitochondria may be implicated in neurodegeneration post-SCI.

In this study, we used three-dimensional morphology to investigate SCI-induced neuroanatomical changes in hippocampal neurons at subcellular structural level following injury, thus impairing the cognitive function. Further, we explored the possible mechanisms behind this process. Importantly, this work provides novel insights into cognitive dysfunction following SCI.

## 2. Materials and methods

### 2.1. Animals and SCI Model Establishment

Adult female Sprague-Dawley (SD) rats (250-300 g) were purchased from the Experimental Animal Center of Ningxia Medical University. All surgical procedures were approved by the Animal Research Ethics Committee of Ningxia Medical University. Rats were maintained in specific pathogen-free (SPF) environment under a 12/12 h light/dark cycle with free access to food and water. After two weeks of acclimatization, animals were randomly divided into four groups: early stage of SCI (1 week post-SCI), late stage of SCI (12 weeks post-SCI), and the week-matched sham controls. A contusion SCI model was established according to previous methods [21]. Briefly, rats were anesthetized with intraperitoneal injection of 1% pentobarbital (5 mg/100 g). After locating T9-T10, the skin, fascia, and paravertebral muscles were incised along the dorsal midline. The vertebral plate and spinous processes were removed with a micro bone biter to expose the spinal cord at T9-T10. SCI was induced by dropping a 10 g weight from a height of 25 cm onto an impactor positioned on the exposed segment. Sham rats only received laminectomy without injuring the spinal cord. The animals were well cared after the surgery. We massaged the animals' bladders three times a day to assist with urination. For rectal dysfunction, we injected enema by a micro hosepipe combined with massage to promote defecation. About 7 to 10 days, the animals could recovery spontaneous urination and defecation. The consistency of the SCI model and postoperative motor function was evaluated by Basso, Beattie, and Bresnahan (BBB) Locomotor Scores [45] at 0, 1, 2, 3, 4, 8, and 12 weeks post-SCI in an open field area.

### 2.2. Three-Dimensional (3-D) Morphological Atlas

#### 2.2.1. Golgi-Cox Staining

Hippocampal Golgi-Cox staining was performed as previously described [46, 47]. Rats were anesthetized with 1% pentobarbital sodium, followed by successive perfusion with 0.9% saline (250 mL, room temperature 20-25°C) and 4% paraformaldehyde solution (500 mL, 4°C). Decapitated and stripped of the skull, the hippocampus was located above the midbrain, extending from front to back and well demarcated from the surrounding brain tissue. Along this limit, the hippocampus was carefully separated with a glass dissecting needle until it was integrally removed. Next, the hippocampus was incubated in Golgi-Cox solution (distilled water 80 g, mercuric chloride 1 g, potassium chromate 0.8 g, potassium dichromate 1 g) and fixed for 60 days at 20-25°C. Subsequently, the hippocampus was immersed in 1% lithium hydroxide, dehydrated in gradient ethanol, ethanol-acetone (1 : 1), acetone, and finally embedded in Spurr resin (SPI, USA).

#### 2.2.2. Fluorescence Micro-Optical Sectioning Tomography (fMOST) Image Processing

To acquire the 3-D morphological atlas of the rat hippocampus, embedded tissue was imaged automatically in an fMOST system (40×, 0.8NA, Wuhan OE-Bio Co., Ltd., China) [48, 49] at a voxel size of 0.3 × 0.3 × 1 *μ*m^3^. Briefly, this system continuously slices along the *Z*-axis with a fixed knife while imaging the surface of the tissue in a Mosaic pattern along the *X* and *Y* axes. The recorded raw image datasets were then integrated and calibrated using a projection curve on an image processing workstation (T7920, Dell Inc., Round Rock, TX, United States) and a computer server (72 cores, 2 GHz per core).

#### 2.2.3. Hippocampus Morphology Reconstruction

We extracted 3-D image blocks (300 × 300 × 300 *μ*m) from each hippocampus and cropped it based on the rat brain atlas. We then converted these raw image blocks into large data access format (LDA). The 3-D neuronal morphologies were reconstructed using the Amira software (Thermo Fisher Scientific, USA), which automatically recognized soma and track fiber projection [50]. We also used the Amira software to generate videos. We measured the dendritic complexity (i.e., the number of dendritic branches and the length of all dendrite fibers) of neurons in 3-D. We also used neuronal synapses as a measure of dendritic complexity.

### 2.3. Immunoblotting

Protein samples were extracted from fresh hippocampal tissue using the Total Protein Extraction Kit (KGP2100, KeyGEN BioTECH, China). Protein concentrations were determined using the BCA Protein Assay Kit (KGPBCA, KeyGEN BioTECH, China). Samples were then electrophoresed by SDS-PAGE and transferred to 0.2 *μ*m PVDF membranes (10600021, Amersham, Germany), which was then cut according to protein marker. Membranes were blocked in 5% bovine serum albumin (BSA) at room temperature for 2 h and incubated in the relevant primary antibodies at 4°C overnight. After washes (3 times 10minutes) in 0.1% tris-buffered saline supplemented with Tween-20 (TBS-T), membranes were incubated in appropriate secondary antibodies at room temperature for 2 h. The protein bands were visualized in an Odyssey Infrared Imaging System (CLX-0796, Gene Company Limited, USA) and quantified with ImageJ software (National Institutes of Health). We used the following antibodies: anti-PSD95 antibody (1 : 2000, ab18258, Abcam, UK), Total OXPHOS Rodent WB Antibody Cocktail (1 : 1000, ab110413, Abcam, UK), Beta Actin Mouse Monoclonal antibody (1 : 5000, 66009-1-Ig, Proteintech, USA), goat anti-mouse (1 : 5000, 926-32210, LI-COR, USA), and goat anti-rabbit (1 : 5000, 926-32211, LI-COR, USA).

### 2.4. Transmission Electron Microscope (TEM)

Rats were anesthetized with 1% pentobarbital sodium and successively perfused with 0.9% saline (250 mL, room temperature 20-25°C) and 4% paraformaldehyde solution (500 mL, 4°C). Subsequently, the hippocampus was separated from the brain and cut into 1 × 1 × 3 mm^3^ pieces under a stereo microscope. Next, the tissue was immersed overnight at 4°C in a mixed solution of 2.5% glutaraldehyde and 2.5% paraformaldehyde. After dehydration and permeation, the tissue was embedded in Spurr resin (SPI, USA) and sectioned. Finally, sections were observed and imaged using a transmission electron microscope (HT7800, Hitachi, Japan).

### 2.5. Reactive Oxygen Species (ROS) Production Assay

Rats were anesthetized and perfused with precooled phosphate buffered saline (PBS) solution. The hippocampus was then separated and prepared into single cell suspensions using mechanical digestion and the trypsin-EDTA solution (T1320, Solarbio, China). The cells were quantified and incubated in H2DCFDA (15 *μ*M, HY-D0940, MCE, USA) or MitoSOX (1 *μ*M, M36008, Thermo, USA) at 37°C for 30 minutes. Finally, the cells were washed with precooled PBS solution and transferred into a fluorescence-activated cell sorting (FACS) tube. The ROS production was measured in a flow cytometer (BD, USA) and analyzed by the FlowJo software (TreeStar Inc.).

### 2.6. Measurement of MDA, SOD, GSH, and ATP

Commercial kits were used to measure SOD activity (BC0175, Solarbio, China), as well as concentration of MDA (BC0025, Solarbio, China), GSH (BC1175, Solarbio, China), and ATP (BC0305, Solarbio, China). Briefly, fresh hippocampal samples were incubated in extraction solutions and completely homogenized on ice. Following centrifugation at 4°C for 10 minutes, the supernatants were collected to perform follow-up tests according to the manufacturer's instructions.

### 2.7. Cognitive Behavioral Tests

Cognitive behavioral tests were performed on week 12 post-injury. Rats were transferred to a quiet and bright behavioral laboratory and allowed to adapt to the environment 30 minutes prior to testing. During testing, behavioral manifestation in each animal was recorded using a video tracking system (SMART 3.0.06, Panlab Harvard Apparatus, Spain).

#### 2.7.1. Morris Water Maze (MWM) Test

The MWM test was performed in a circular tank filled with black water, which was divided into four quadrants. A platform was placed below the water level in a constant quadrant for animals to escape. The rats were placed in the water from each quadrant and left to find the hidden platform for 4 consecutive training days. If the animal was unable to find the platform within 60 seconds, it would be guided to the platform with a crabstick and allowed to stay on the platform for 10 seconds. On the fifth day, the platform was removed, and the rats were allowed to move freely for 60 seconds. Latency to platform, platform area crossings, and MWM search strategy were recorded for further analysis as previously described [20, 21, 51].

#### 2.7.2. Open Field (OF) Test

The OF test was performed using an open-field chamber divided into a central zone and a marginal zone. Animals were individually placed in the center of the field and allowed to explore freely for 10 minutes. The traveling distances of central/marginal zone were recorded. The proportion of the distance in central zone was defined as (distance in central/[distance in central + distance in marginal] × 100%). After a test had been completed, the chamber was thoroughly cleaned by 75% ethanol to suppress interference between individual animals.

#### 2.7.3. Elevated Plus-Maze (EPM) Test

For EPM tests, we used an elevated plus-shaped maze, which consisted of a central zone, two open arms, and two closed arms with side walls. When the test began, animals were individually placed in the central zone to allow to explore the maze for 5 minutes. The proportion of distance travelled and entries in open arms were calculated as (open arms/[open arms + closed arms] × 100%). Again, the apparatus was cleaned with 75% ethanol between tests.

#### 2.7.4. Three-Chamber Social Interaction (TCSI) Test

In this test, the apparatus consisted of three rectangular chambers. Two cylindrical cages were placed in two lateral chambers, which connected the central chamber with a small door. The test was divided into three sessions. Session 1: a model rat freely explored the central chamber and two empty lateral chambers for 10 minutes to habituate the apparatus. Session 2: an unfamiliar rat (stranger 1-S1) was placed in one of the cylindrical cages located in a lateral chamber, while another cage was empty (empty-*E*). The rat from session 1 freely explored three chambers for 10 minutes. Session 3: another unfamiliar rat was placed the empty cage (stranger 2-S2) of session 2, and the same rat was allowed to explore three chambers for 10 minutes again. The time spent in close proximity with stranger 1, empty, and stranger 2 was recorded and analyzed as previously described [52, 53] with some modifications. The preference index for each rat spent in close proximity was calculated as (S1/[S1 + *E*] × 100%) or (S2/[S1 + S2] × 100%).

### 2.8. Statistical Analysis

All data were analyzed in GraphPad Prism 6.01 (GraphPad Software, San Diego, CA, USA) and are presented as the mean ± standard deviation (SD). Unpaired two-tailed Student *t*-tests or chi-square tests were applied for two group comparisons. Two-way analysis of variance (ANOVA) with a posthoc test was applied for multigroup comparisons. Statistical significance was set to *P* values <0.05.

## 3. Results

### 3.1. SCI Led to Long-Term Deficits of Motor Function

**Figure 1:**
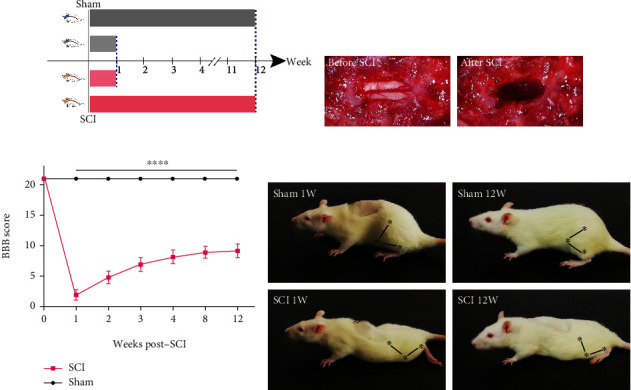
SCI led to long-term deficits of motor function. (a) Grouping scheme. (b) Before and after SCI. (c) Motor function deficits after SCI were assessed by BBB scores over 12 weeks. (Data were presented as means ± SD, *n* = 8, Student's unpaired *t*-test at different time points, ^∗∗∗∗^*P* < 0.0001, sham vs. SCI). (d) Representative motor deficits of each group. The symbols represent joints (the hip, knee, and ankle joints).

To determine whether the effects of SCI on the hippocampus were progressive, we divided the animals into four groups (Figure 1(a)): early stage of sham (1 week after Sham), early stage of SCI (1 week after SCI), late stage of sham (12 weeks after sham), and late stage SCI (12 weeks after SCI). We observed a marked hematoma at the site of injury after SCI (Figure 1(b)). Subsequently, we used the BBB score to evaluate motor function at different time points. The results showed that although the animals gradually recovered, SCI led to long-term deficits of motor function (Figures 1(c) and 1(d); all *P* < 0.0001). The long-term deficit from the local injured spinal cord could have a negative cumulative effect on brain areas, that are directly or indirectly connected to the injured spinal cord.

### 3.2. SCI Accelerated Oxidative Stress Damage of Hippocampal Neuronal Mitochondria

**Figure 2:**
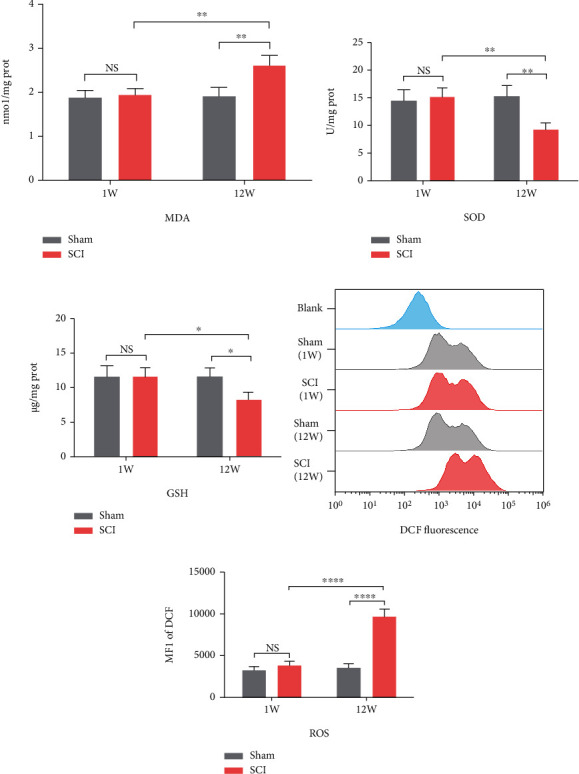
SCI accelerated oxidative stress in hippocampus. (a)–(c) MDA concentrations, SOD activity, and GSH concentrations in the hippocampus were determined according to the respective assay kit. (d, e) Level of ROS in hippocampus was assessed by flow cytometry using H2DCFDA (all data were presented as means ± SD, *n* = 4, two-way ANOVA, NS: no significance, ^∗^*P* < 0.05, ^∗∗^*P* < 0.01, and ^∗∗∗∗^*P* < 0.0001).

**Figure 3:**
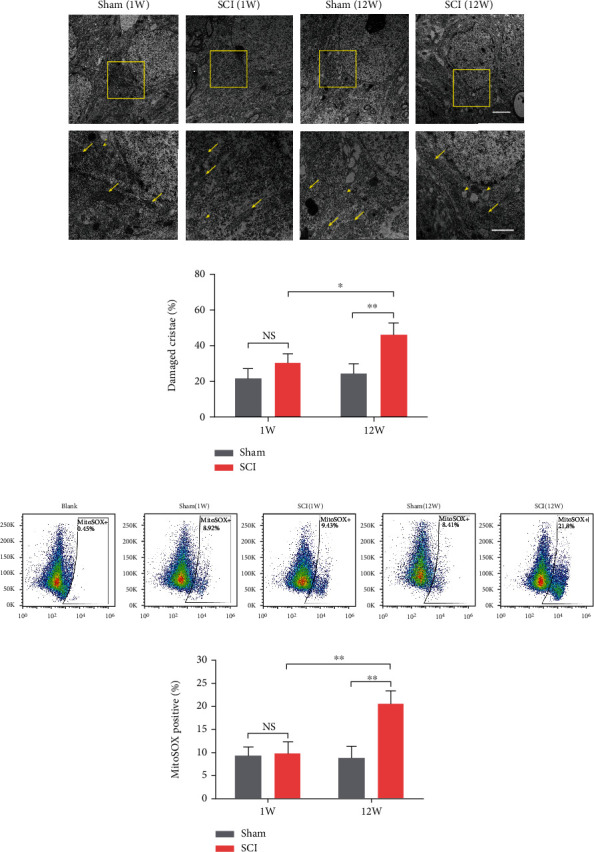
High levels of ROS induced mitochondrial damage in hippocampal neurons. (a, b) The mitochondrial structures of hippocampal neurons were observed by (TEM). Arrows, normal mitochondria. Arrowheads, damaged mitochondria. Scale bars = 2 *μ*m (above) or 1 *μ*m (below). (c, d) Mitochondrial ROS was assessed by flow cytometry using MitoSOX (data were presented as means ± SD, *n* = 3, two-way ANOVA, NS: no significance, ^∗^*P* < 0.05, ^∗∗^*P* < 0.01).

**Figure 4:**
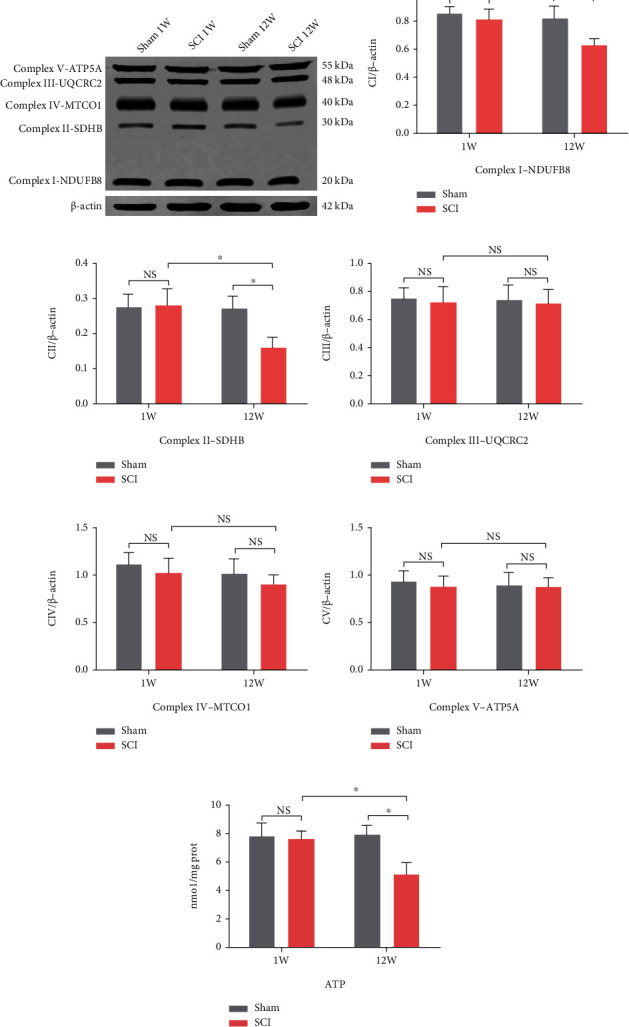
Mitochondrial function was assessed by respiration chain and ATP production. (a)–(f) Representative western blot bands and quantitative analyses of oxidative phosphorylation (OXPHOS) complexes. (g) ATP concentration was tested according to the assay kit (data were presented as means ± SD, *n* = 3, two-way ANOVA, NS: no significance, ^∗^*P* < 0.05).

Oxidative stress plays an important role in neurodegeneration. To evaluate the local oxidative levels in the hippocampus after SCI, we measured the SOD activity, MDA, and GSH concentrations of each group. The MDA concentrations in the SCI (12 W) group were significantly higher than in the SCI (1 W) and sham (12 W) groups (*P* = 0.0017 and *P* = 0.0012, respectively; Figure 2(a)). SOD activity and GSH concentrations in the SCI (12 W) group decreased significantly compared to the SCI (1 W) group (*P* = 0.0016 and *P* = 0.0151, respectively) and the sham (12 W) group (*P* = 0.0013 and *P* = 0.0154, respectively) (Figures 2(b) and 2(c)). Moreover, there were no significant differences between the sham (1 W) and SCI (1 W) groups of in terms of MDA concentrations (*P* > 0.05), SOD activity (*P* > 0.05), and GSH concentrations (*P* > 0.05). ROS is the major contents of oxidative stress. As expected, we observed ROS accumulation in SCI (12 W) rats (*P* < 0.0001 vs. SCI (1 W) and sham (12 W)). One week after SCI, accumulation of ROS was not significant (*P* > 0.05) (Figures 2(d) and 2(e)). Using TEM to study the mitochondrial structure of hippocampal neurons, we identified large numbers of damaged mitochondrial cristae in the SCI (12 W) group (*P* = 0.0337 vs. SCI (1 W) and *P* = 0.0058 vs. sham (12 W)) (Figures 3(a) and 3(b)). Next, we measured mitochondrial ROS using MitoSOX, a selective mitochondrial ROS indicator. Here, we found that mitochondrial ROS were higher in the SCI (12 W) group compared to the SCI (1 W) group (*P* = 0.003) and sham (12 W) animals (*P* = 0.0017; Figures 3(c) and 3(d)). Further, we investigated mitochondrial function by mitochondrial respiratory chain and ATP production. Interestingly, we observed downregulation of Complex I-NDUFB8 and Complex II-SDHB expression in SCI (12 W) animals (*P* = 0.0482, *P* = 0.0291 vs. SCI (1 W) and *P* = 0.0416, *P* = 0.0417 vs. sham (12 W)) (Figures 4(a)–4(c)). Complex III-UQCRC2, Complex IV-MTCO1, and Complex V-ATP5A expression were not significantly different (all *P* > 0.05; Figures 4(a), 4(d), 4(e), and 4(f)). Unsurprisingly, ATP production was also reduced in the SCI (12 W) group (*P* = 0.0226 vs. SCI (1 W) and *P* = 0.0124 vs. sham (12 W) (Figure 4(g)). These findings suggest that accelerated oxidative stress results in mitochondrial abnormalities and decreased ATP production in late stage of SCI.

### 3.3. Mitochondrial Abnormalities Induced the Reduction of Dendritic Complexity and Synaptic Communication

**Figure 5:**
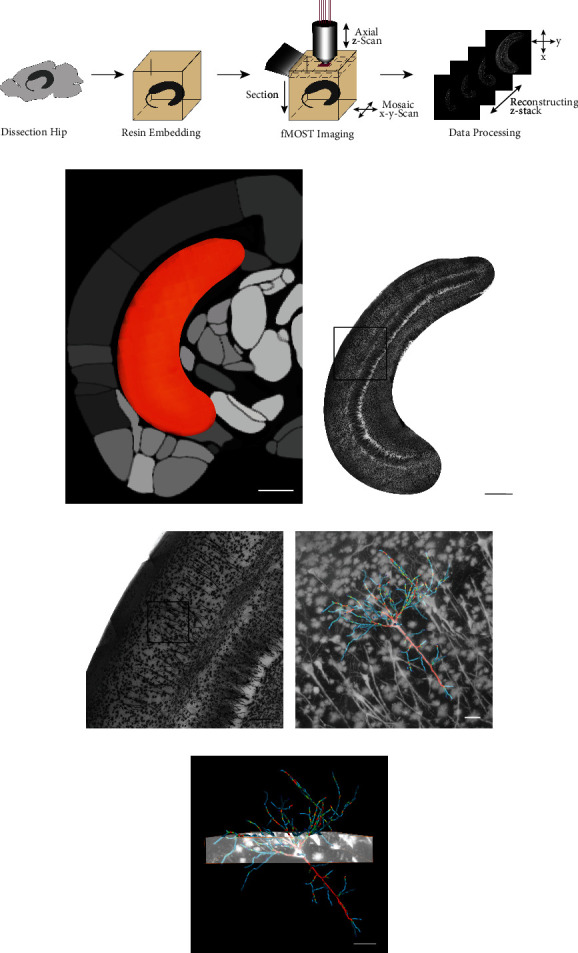
3-D morphological atlas. (a) Diagram illustrating the fMOST system. (b) The hippocampus pseudocolor image of the fMOST system in coronal map of rat brain. Scale bars = 1500 *μ*m. (c) The entire projection image of hippocampal neurons (Video 1). Scale bars = 1000 *μ*m. (d) Enlarged hippocampal neurons projection image. Scale bars = 200 *μ*m. (e, f) Processes of 3-D reconstruction, including data set import, soma location, projection tracing, and date block clipping (Video 2). Scale bars = 50 *μ*m.

**Figure 6:**
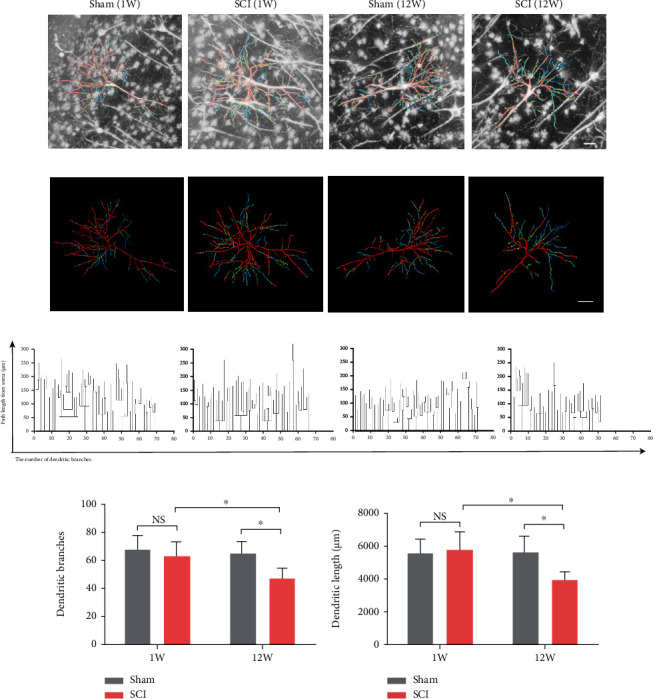
Dendrite complexity of hippocampal neurons was reduced in late stage of SCI (12 weeks after SCI). (a) Representative date block and (b) 3-D reconstructed neurons of each group (Video 3). (c) Respective dendrograms showed the length and branches of neurons. (d, e) Quantitative analysis of the total dendritic length and branches (data were presented as means ± SD, *n* = 6 from 3 rats, two-way ANOVA, NS: no significance, ^∗^*P* < 0.05). Scale bars = 50 *μ*m.

To investigate dendritic complexity, we generated a 3-D morphological atlas of rat hippocampal neurons using the fMOST system. The imaging process involved four main steps: dissection of the hippocampus, resin embedding, fMOST imaging, and data processing (Figure 5(a)). The hippocampus pseudocolor image from the fMOST system matched well with coronal map of rat brain (Figure 5(b)). The localization of the entire hippocampus neuronal soma and dendritic projections could be clearly distinguished (Figures 5(c) and 5(d) and Video 1). The boundaries of the pyramidal, molecular, and polymorphic layers were distinct, but they were connected by dendritic projections. Further, we reconstructed pyramidal neurons including: data set import, soma location, projection tracing, and date block clipping (Figures 5(e) and 5(f) and Video 2). The reconstructed neurons displayed a typical pyramidal morphology with extensive dendrites and central soma. Representative date block and reconstructed neurons for each group are shown in Figures 6(a) and 6(b). Owing to the availability of precise whole hippocampal data sets, the excellent dendritic morphology was presented integrally (Video 3). Dendritic complexity was measured using dendrograms (Figure 6(c)), which illustrated the overall branching pattern of the dendrite tree via length and branches. The total branches and lengths of dendrites were reduced in the SCI (12 W) group compared to the SCI (1 W) (*P* = 0.0346 and *P* = 0.0149, respectively) and sham (12 W) groups (*P* = 0.0156 and *P* = 0.0316, respectively) (Figures 6(d) and 6(e)). However, there were no significant differences between sham (1 W) and SCI (1 W) animals (*P* > 0.05).

**Figure 7:**
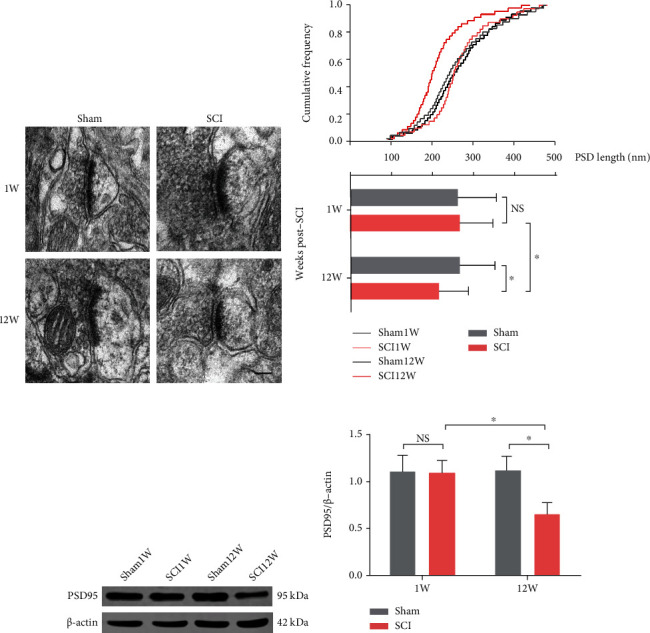
Synaptic communication was decreased in late stage of SCI. (a) Representative electron micrographs of PSD. Scale bars = 100 nm. (b) The cumulative frequency and quantitative analysis of PSD length (data was presented as means ± SD, *n* = 40 − 45, from 3 rats, two-way ANOVA, NS: no significance, ^∗^*P* < 0.05). (c, d) Representative western blot bands and quantitative analyses of PSD95 (data were presented as means ± SD, *n* = 3, two-way ANOVA, NS: no significance, ^∗^*P* < 0.05).

We further investigated synaptic communication by TEM and western blot. We found that the postsynaptic density (PSD) was significantly shorter in the SCI (12 W) group compared to the SCI (1 W) group (*P* = 0.0303) and the sham (12 W) group (*P* = 0.0187; Figures 7(a) and 7(b)). Moreover, we observed downregulation of the PSD95 protein expression in the SCI (12 W) group compared to SCI (1 W) (*P* = 0.0372) and sham (12 W) animals (*P* = 0.0282; Figures 7(c) and 7(d)).

### 3.4. Cognitive Function Was Impaired after SCI

We used MWM, OF, EPM, and TCSI tests to evaluate cognitive function. To minimize the effects of motor deficits on the tests themselves, we performed these tests in late stage of SCI and compared the data in percentage terms.

**Figure 8:**
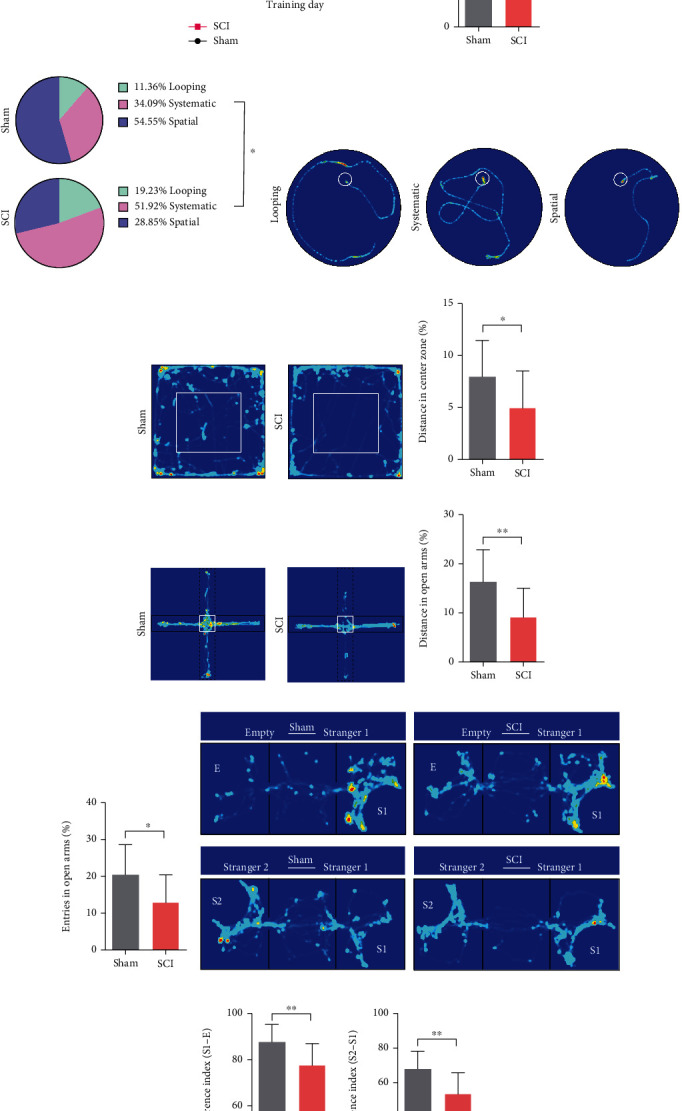
Cognitive impairments after SCI. (a) The latency to platform for 4 consecutive training days in MWM test. There was a significant difference between sham and SCI groups on day 4 (data were presented as means ± SD, *n* = 11 sham, *n* = 13 SCI, Student's unpaired *t*-test, ^∗∗∗∗^*P* < 0.0001). (b) The total platform crossings on day 5 of MWM test (data were presented as means ± SD, *n* = 11 sham, *n* = 13 SCI, Student's unpaired *t*-test, ^∗∗^*P* < 0.01). (c) The search strategy in MWM test (data were presented as constituent ratio, *n* = 11 sham, *n* = 13 SCI, chi-square test, ^∗^*P* < 0.05). (d) Representative heat map of search strategies. (e-f) Representative heat map in OF test and the proportion of distance travelled in the central zone (%). (Date were presented as means ± SD, *n* = 11 sham, *n* = 13 SCI, Student's unpaired *t*-test, ^∗^*P* < 0.05). (g)–(i) Representative heat map in EPM test, the proportion of distance travelled in open arms (%), and entries in open arms (%) (data were presented as means ± SD, *n* = 13 Sham, *n* = 12 SCI, Student's unpaired *t*-test, ^∗∗^*P* < 0.01, ^∗^*P* < 0.05). (j)–(l) Representative heat map of TCSI test, preference index of “Stranger 1—Empty” and “Stranger 2—Stranger 1.” (data were presented as means ± SD, *n* = 13 sham, *n* = 12 SCI, Student's unpaired *t*-test, ^∗∗^*P* < 0.01).

In MWM test, SCI rats exhibited prolonged latency to the platform compared to sham rats at training days (*P* < 0.0001 at day 4; Figure 8(a)). When the platform was removed at day 5, SCI rats achieved fewer platform crossings compared to sham rats (*P* = 0.0085; Figure 8(b)). To eliminate the confounding effect caused by swimming speed, we implemented 3 search strategies to evaluate the efficiency of platform positioning, which was less affected by motor deficits [21, 51]. There were significant differences between sham (looping 11.36%, systematic 34.09%, spatial 54.55%) and SCI rats (looping 19.23%, systematic 51.92%, spatial 28.85%) (*P* = 0.0378; Figure 8(c)). The representative heat map of the search strategies is shown in Figure 8(d). These data suggested that spatial learning and memory were impaired following SCI.

OF tests were used to examine spontaneous exploring activity. SCI rats showed a decreased preference for moving into the central zone compared to sham rats (*P* = 0.0483; Figures 8(e) and 8(f)). EPM test is based on the observation that rats have an inherent preference for the closed arms of the maze rather than the open arms. SCI rats exhibited a larger distance (*P* = 0.0079) and more entries (*P* = 0.019) in open arms (Figures 8(g), 8(h), and 8(i)).

Furthermore, the rats were tested for voluntary social interaction and discriminatory behavior using TCSI test. The representative heat map in Figure 8(j) illustrates the performance of sham and SCI rats in this test. Both groups showed a preference for a social partner (“S1”) rather than an empty cage. However, the preference index for SCI rats was significantly lower (*P* = 0.0069) (Figure 8(k)). When another unfamiliar novel social partner (“S2”) was placed in the empty cage, the SCI rats showed a lower preference index for this novel stimulus (*P* = 0.004) (Figure 8(l)). The differences in preference between these two groups may result from both social recognition deficits, compounded by short-term memory impairment. Taken together, our data suggest that cognitive function was impaired after SCI.

## 4. Discussion

**Figure 9:**
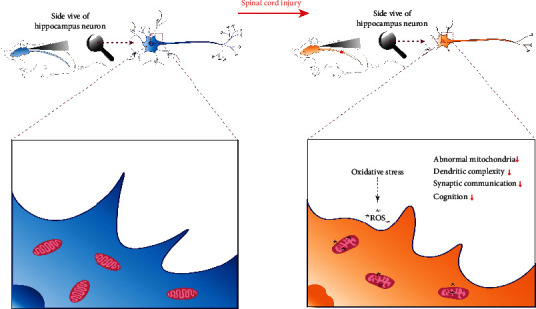
Proposed mechanism of cognitive impairments following SCI. SCI accelerated oxidative stress damage of hippocampal neuronal mitochondria, resulting in a reduction of dendritic complexity and synaptic communication, leading to cognitive decline.

The large-scale and multistage of the central nervous system determined that the effects of SCI were not only limited to the local site of injury but were also spread to most of the brain functional areas, which were involved in genetic, environmental, and endogenous factors. Differential protein expression, imbalanced oxidative stress, and mitochondrial dysfunction, as well as changes in neurotransmitter and neuroinflammatory factors, have been suggested as potential pathophysiological mechanisms behind SCI. Here, we demonstrated for the first time that SCI accelerated oxidative stress in hippocampus, resulting in ROS damage to neuronal mitochondria, leading to reduced dendrite complexity and synaptic communication, which may eventually lead to cognitive impairment (Figure 9).

Oxidative stress is closely related to neurodegenerative pathology. Oxidative stress, characterized by excessive ROS production, can lead to mitochondrial damage via different routes, including mutations in mitochondrial DNA, disrupted respiratory chain activity, changes in membrane permeability, and altered Ca^2+^ homeostasis [54]. Most previous studies focused on chronic inflammation in the brain after SCI. Aberrant activation of microglia has been observed during brain degeneration following SCI [21]. Microglial depletion significantly reduced chronic neuroinflammation in the brain and improved neurological recovery after SCI, including depressive-like behavior and cognition function [25]. Importantly, inflammation and oxidative stress are mutually reinforcing. Further, oxidative stress is viewed as an imbalance between oxidation and antioxidant systems, which may activate a variety of transcription factors involved in inflammatory pathways [55]. Although studies have reported chronic inflammation in hippocampus after SCI, the involvement of oxidative stress has not been proven. In this study, we found that accelerated oxidative stress resulted in increased levels of ROS in hippocampus and increased mitochondrial damage in late stage of SCI. These structurally damaged mitochondria were dysfunctional and unable to produce sufficient ATP for neurons to maintain normal physiological activities. A previous study using a rat model of SCI demonstrated ROS accumulation, aberrant mitochondria, and neuronal loss in the motor cortex [56]. In addition, a recent publication showed that restore mitochondrial function by some therapeutic approaches could delay the onset and slow the progression of Alzheimer's disease [57].

In modern neuroscience research, the position of neurons and neural circuits is usually defined by relatively simple two-dimensional (2-D) brain maps. Changes in neuronal conformation and neural circuit remodeling are also typically analyzed by 2-D pathological sections. Neurons with different functions present different sizes, shapes, and at different positions, and even neighboring neurons of the same cell type differ in morphology and projection patterns [50]. However, obtaining high-throughput information from a complex biological system while maintaining its integrity is a significant challenge in neuroscience research. The 3-D and stereoscopic nature of the organization determines that researches based on 2-D plane might inevitably miss some important information. Additionally, spatial differences at the cellular level may lead to localization errors and an inability to analyze neuronal morphology at single-cell resolution. Novel technologies have emerged in recent years to address these challenges, thereby allowing us to study morphological features based on 3-D models, which mainly display the 3-D conformation of tissue structure through physical or chemical methods, for example, CLARITY [58], uDISCO [59], and fMOST system [48]. In this study, we obtained a 3-D neuroanatomical atlas of rat hippocampal neurons at the subcellular structural level using the fMOST system. To our knowledge, this is the first 3-D morphological map of the rat hippocampus in which the different layers of neuronal soma location and dendritic projection could be clearly distinguished. Subsequently, we reconstructed pyramidal neurons to analyze dendritic complexity. The data showed that dendritic complexity was reduced with fewer dendritic branches and shorter dendrites in late stage of SCI. However, there were no significant differences in early stage of SCI. Similar results were found in animal models of Alzheimer's disease [60, 61]. The observed changes in dendritic complexity further strengthen our hypothesis regarding synaptic communication. Therefore, we performed additional morphological and biochemical analyses focusing on PSD, whose structure and composition usually serve as an indicator of synaptic communication. In our experiments, the PSD faced the presynaptic active region, thus receiving most of the excitatory synaptic input and promoting transduction of postsynaptic signaling [62]. In line with dendritic complexity, TEM data suggested a significant reduction in PSD length. In addition, western blot analyses also demonstrated a significant downregulation of the PSD95 protein expression. These results show that SCI induced a loss in dendritic complexity and synaptic communication in the hippocampus.

Cognitive and mood disorders have consistently been observed in patients with SCI and in experimental models of SCI [28]. SCI-induced effects on cognitive function are extremely complex. The spinal cord contains a mass of ascending and descending fasciculus, which directly or indirectly connected to the different brain regions. SCI may cumulatively impact these regions at the local site of injury, thus initiating progressive neurodegeneration. Here, we used a series of behavioral tests to evaluate cognitive functions. It is important to note that motor deficit may cause potential confounding effects in the behavioral tests. We took the following actions to address these effects: (i) we employed 4 different tests, three of which (the OF, EPM, and TCSI) were less affected by motor deficits; (ii) we compared the results of these three tests in percentage terms to eliminate the effect of total distance or time of movement in the tests; (iii) we performed three search strategies to evaluate the efficiency of platform positioning in the MWM test, which provides complementary outcome measures that might be less dependent on motor function. This method has been widely used to evaluate cognitive function after SCI and traumatic brain injury in rodents, both of which could lead to a deficit in motor function [20, 21, 51]; (iv) we executed these tests in late stage SCI when partial motor function was recovered. We observed that SCI rats were able to successfully complete the tests. The MWM test has been commonly used to evaluate spatial learning and memory. SCI rats exhibited prolonged latency to platform and decreased platform crossings. Compared to sham rats, SCI rats showed decreased spatial strategy, increased looping, and systematic strategy. We continued to evaluate spontaneous exploring and anxiety-like behavior after SCI. Like the MWM test, SCI rats performed poorly in OF and EPM tests. We also evaluated hippocampus-dependent voluntary social interaction and discriminatory behavior using the TCSI test. SCI rats showed a lower preference for social partner 1 and novel partner 2. Taken together, these data suggest impairment of cognitive function post-SCI.

In summary, the present study suggests that the effects of SCI on the brain are progressive. Cognitive impairment is not just caused by environmental factors but rather specific neuroanatomical changes caused by abnormal mitochondria. Interventions that target these abnormalities may offer further detailed insights into the biological mechanisms involved. Antioxidant therapy may be a promising therapeutic strategy.

## Data Availability

The data used to support the findings of this study are available from the corresponding author upon request.

## Supplementary Materials

Supplementary Materials Video 1: the entire hippocampus neuronal soma location and dendritic projection. Video 2: the processes of 3D reconstruction pyramidal neuron. Video 3: the representative date block and 3D reconstructed neurons of each group. (https://drive.google.com/file/d/1NtwHdVBEXIzNnHW54gvf61RJRz8eo3Ev/view?usp=sharing).
