# Supplementary material for: Hippocampal Mitochondrial Abnormalities Induced the Dendritic Complexity Reduction and Cognitive Decline in a Rat Model of Spinal Cord Injury
Source: Oxid Med Cell Longev. 2022 May 4;2022:9253916. doi: 10.1155/2022/9253916 (PMC9095360; doi:10.1155/2022/9253916)
Supplement: Supplementary Materials — Video 1: the entire hippocampus neuronal soma location and dendritic projection. Video 2: the processes of 3D reconstruction pyramidal neuron. Video 3: the representative date block and 3D reconstructed neurons of each group. (https://drive.google.com/file/d/1NtwHdVBEXIzNnHW54gvf61RJRz8eo3Ev/view?usp=sharing). [file 9253916.f1.docx]

**Supplementary materials (Videos)**

**Manuscript title**

“Hippocampal Mitochondrial Abnormalities Induced the Dendritic Complexity Reduction and Cognitive Decline in a Rat Model of Spinal Cord Injury”.

Video 1: The entire hippocampus neuronal soma location and dendritic projection.

Video 2: The processes of 3D reconstruction pyramidal neuron.

Video 3: The representative date block and 3D reconstructed neurons of each group. (<https://drive.google.com/file/d/1NtwHdVBEXIzNnHW54gvf61RJRz8eo3Ev/view?usp=sharing>).
